# Next-Generation Sequencing for the Detection of Microbial Agents in Avian Clinical Samples

**DOI:** 10.3390/vetsci10120690

**Published:** 2023-12-04

**Authors:** Claudio L. Afonso, Anna M. Afonso

**Affiliations:** 1 BASE2BIO, 1945 Arlington Drive, Oshkosh, WI 54904, USA; 2 Independent Researcher, Athens, GA 30601, USA

**Keywords:** NGS, diagnostics, avian, chicken, surveillance, random sequencing, targeted sequencing, clinical, viruses, bacteria, bioinformatics

## Abstract

**Simple Summary:**

Significant progress in next-generation sequencing (NGS) is positioning this technology as a key tool to be utilized in clinical diagnosis of disease agents and/or for veterinary surveillance. Recent advances in direct sequencing of poultry and other avian samples for the detection of microbial agents are reviewed here. This review, although not comprehensive, highlights key developments in avian NGS-based technology for diagnostic uses during the last five years and discusses the future challenges for practical implementation, as well as potential applications in new areas related to poultry production.

**Abstract:**

Direct-targeted next-generation sequencing (tNGS), with its undoubtedly superior diagnostic capacity over real-time PCR (RT-PCR), and direct-non-targeted NGS (ntNGS), with its higher capacity to identify and characterize multiple agents, are both likely to become diagnostic methods of choice in the future. tNGS is a rapid and sensitive method for precise characterization of suspected agents. ntNGS, also known as agnostic diagnosis, does not require a hypothesis and has been used to identify unsuspected infections in clinical samples. Implemented in the form of multiplexed total DNA metagenomics or as total RNA sequencing, the approach produces comprehensive and actionable reports that allow semi-quantitative identification of most of the agents present in respiratory, cloacal, and tissue samples. The diagnostic benefits of the use of direct tNGS and ntNGS are high specificity, compatibility with different types of clinical samples (fresh, frozen, FTA cards, and paraffin-embedded), production of nearly complete infection profiles (viruses, bacteria, fungus, and parasites), production of “semi-quantitative” information, direct agent genotyping, and infectious agent mutational information. The achievements of NGS in terms of diagnosing poultry problems are described here, along with future applications. Multiplexing, development of standard operating procedures, robotics, sequencing kits, automated bioinformatics, cloud computing, and artificial intelligence (AI) are disciplines converging toward the use of this technology for active surveillance in poultry farms. Other advances in human and veterinary NGS sequencing are likely to be adaptable to avian species in the future.

## 1. Introduction

Past reviews describe recent progress and future perspectives on the use of next-generation sequencing (NGS) in veterinary medicine [1,2,3,4,5,6,7,8,9,10]. Key advances in mammalian NGS-based diagnostics include the adoption of different types of sequencing platforms and the development of specific applications of NGS for the different mammalian hosts common for human and veterinary medicine. These advances will not be discussed here, except when necessary to highlight the potential of those technologies in poultry diagnostics. Here, we will focus on the advances of NGS for avian and poultry medicine during the last few years. This review, which is not comprehensive, intends to highlight key NGS diagnostic developments, limitations, challenges, and potential future applications of NGS in avian medicine and poultry meat production.

## 2. Direct-Targeted and Non-Targeted NGS versus Classical Diagnostics

There is an evident need for the rapid genetic characterization of host pathogens in order to facilitate healthy production and for the characterization of genetic changes in RNA viruses that threaten poultry health. Diseases caused by these viruses, such as Newcastle disease virus (NDV), infectious bronchitis virus (IBV), and infectious bursal disease virus (IBDV), are often controlled using live vaccines. In those cases, the disease-causing and the vaccine viruses can multiply simultaneously and co-exist inside one single animal (or farm), making specific diagnosis difficult. Furthermore, the high frequency of mutation and/or recombination of RNA viruses accentuates the limitations of classical diagnostics, such as those performed using the RT-PCR technique. RT-PCR has the double disadvantage of providing no genetic information on the agent identified and of being adversely sensitive to mutations at the primer or probe sites used for the tests. In the past, pathogen genetic information has been acquired, and mutation analysis of pathogens has been achieved, through sequencing with the targeted Sanger method as a second step, following initial detection via PCR or other classical diagnostic approaches such as ELISA, immunohistochemistry, virus growth tests, agglutination assays, microscopy, or electron microscopy. However, new NGS-based technologies promise to provide low-cost alternatives that may allow the utilization of tNGS and ntNGS as the first line of diagnostics. As multiplexing technologies continue to improve and the cost of sequencing continues to drop, targeted and non-targeted direct sequencing are likely to become desirable diagnostics methodologies capable of replacing RT-PCR. Thus, an initial comprehensive NGS analysis of nucleic acids for effective pathogen detection directly on clinical samples is likely to become a common tool used to enhance the prevention and control of avian diseases, improving poultry production.

Quite often, the identification of a single agent using classical diagnostics does not immediately lead to a problem in a field being solved. Slow and limited binary (positive or negative) targeted diagnostics tests have dominated classical veterinary diagnostics for years. A not-so-recognized fact is that, in most farms and geographic locations, more than one pathogen or variants of a pathogen (and in some cases opportunistic pathogens) are co-circulating and affecting productivity [11,12]. Co-infections and interactions between pathogens have always been suspected of being present in farm animals, but the ability to rapidly characterize multiple agents for the development of effective control strategies is a recent development [13,14,15]. In mammalian and avian mucosal tissues, the presence of more than one viral disease-causing agent is common, and the coexistence of viruses and bacteria (or other eukaryotic disease-causing agents) is almost guaranteed [16,17,18,19,20]. While a complete classic diagnostic panel or a combination of classical tests may be useful, as it has often been implemented in human medicine, it is in many cases not economically feasible to routinely perform complete classical diagnosis of all suspected disease agents during active surveillance of chickens or other bird species. However, it is reasonable to imagine that a complete analysis of all the microbiological content of respiratory, cloacal, and immune-related tissues could help in controlling diseases and/or improving nutrition in poultry if cost were not a factor.

ntNGS (also known as random NGS) of nucleic acids in diagnostic samples have the potential to characterize complex infections with greater speed than traditional diagnosis and, in turn, lead to more effective prevention and control of poultry diseases. Additionally, periodic ntNGS analysis of circulating agents in respiratory, cloacal, and immune tissues at the farm level should help veterinarians and managers to understand the complex issues associated with efficient production to make accurate and reasonable treatment/management decisions. Comprehensive NGS-based diagnostics can also be used to support and complement the current hypothesis-based (positive–negative) type of approach used in classical diagnostics. For example, a preliminary detection of circulating agents in a dead animal using ntNGS could precede the use of cheaper specific tests based on the use of antibodies or RT-PCR in the entire flock (or an entire geographic area). These two are valuable and useful classical low-cost diagnostics tools that may confirm an ntNGS initial characterization. Individual PCR or ELISA are the most used tests to provide low-cost diagnostics limited to a single agent. However, most of these classical techniques have the disadvantage that they require the existence of a previous hypothesis and the use of agent-specific reagents. Thus, hypothesis-based classical diagnostics, although fast and accurate for detecting the presence of single agents, can be slower and more expensive for precisely characterizing unknown agents or for detecting mixed infections. Understanding farm problems often not only involves agent detection, but also requires the obtention of precise genetic information, mutation identification, detection of co-infecting and opportunistic disease-causing agents, and in some cases an analysis of the host response. This additional information can be provided on a first instance via direct ntNGS.

ntNGS is now advancing as a powerful tool for farm-animal diagnostics [21,22,23,24,25] because it can identify and simultaneously genetically characterize multiple agents for each sample. Technical and logistic challenges are rapidly being resolved and one can envision that it will not be long before every farm will periodically obtain a report providing the “microbiological status” of the farm based on random or non-targeted NGS analysis. Progress in obtaining considerably deeper and informationally richer outputs, coupled with advances in robotics and multiplexing (to simultaneously process and reproducibly sequence multiple samples) and automated bioinformatic analysis during the last five years, is moving this technology out of the research lab and into new field applications. For example, recently NGS has been used as a powerful tool for the active surveillance of poultry farms across South America with the potential to provide information useful for flock management and for selection of more specific vaccines [15,25].

## 3. Short-Read Sequencing on Poultry and Avian Species

Currently, two basic sequencing approaches dominate NGS applications. Short- and long-read sequencers represent these techniques. Historically, short-read sequencers (e.g., Illumina, https://www.illumina.com/, accessed on 24 November 2023) have provided more accurate sequences but have the disadvantage that the assembly of sequences is sometimes more difficult to perform because nucleic acids are broken down into smaller fragments. Most short-read sequencing applications are implemented using high- or medium-throughput machines, although some small sequencers in the market may become mainstream in the future. A low-throughput short-read sequencer is being offered by Illumina, although it has not proven yet to produce outputs of comparable quality to other higher-throughput machines (https://www.illumina.com/systems/sequencing-platforms/iseq/specifications.html, accessed on 24 November 2023). Recently, several reports have indicated that, in the near future, other possibly competitive technologies may offer new applications and cost-effective advantages that could be game changers for the utilization of the direct sequencing of clinical samples as diagnostics tools (https://www.science.org/content/article/100-genome-new-dna-sequencers-could-be-game-changer-biology-medicine, accessed on 24 November 2023).

The core technology most used in short-read NGS has been around for many years, although iterative improvements have been made in cost, read length, and throughput. Consequently, numerous short-read sequencing diagnostics applications have been developed at the laboratory level. Early applications of random sequencing were developed for the genetic characterization of NDV using formalin-fixed paraffin-embedded (FFPE) samples [26,27,28,29]. Short-read platforms were used for non-targeted genetic characterization and the discovery of unsuspected agents in paraffin-embedded samples from pigeons and chickens. FFPE tissue samples, which are routinely used to provide classical pathogenesis diagnostics, are widely available in pathology labs used to perform NGS studies. In addition, these are also often used to conduct retrospective studies on old cases because of the stability and safety of the fixed samples and the ease of transport. He et al. used stored archival FFPE samples from wild pigeon spleen, kidney, and liver samples obtained from previous mortality events in the U.S. [28]. Total RNAs were extracted from FFPE sections and a protocol was developed to sequence total RNAs using a non-targeted approach. When results were compared with immunohistochemistry (IHC), a more complete understanding of the extent of damage caused by NDV on wild pigeon’s tissues was obtained as it was possible to relate tissue damage to virus presence. Interestingly, most NGS-positive samples produced sufficient nucleic acids to obtain an average genome coverage of > 99%. The sequence allowed the identification of viruses as members of a previously described sub-genotype (VIa) or novel sub-genotype (VIn) of NDV. Results demonstrated that all the different types of tissues extracted were suitable for RNA extraction and for sequencing, with 100% identity among samples collected from different tissues. In all cases, the deduced amino acid cleavage site of the fusion protein, which is normally used to identify virulent NDV, was obtained. When the sequence from the USDA-validated real-time RT-PCR assay primers and probe tests (normally used to detect virulent NDV) was compared to sequences obtained from pigeons via NGS, two to four mismatches were found in each of the oligonucleotides. This demonstrated that, while RT-PCR had the potential for failure or reduced sensitivity, random sequencing of the same samples could detect emerging mutations and determine the potential virulence of the agent. Since most of the samples were archival specimens, some sampled 2 to 7 years ago, the viability of the approach for use with years-old archival tissue samples was also demonstrated.

Butt extended the applicability of NGS in fixed samples to obtain the complete genome analysis of NDV and to closely track the evolution of very related viruses [29]. FFPE samples of the spleens, lungs, brains, and small intestines of chickens infected during an outbreak were safely shipped from farms in Pakistan to the US and used to extract total RNA. The samples originated from different poultry flocks during a short period of an outbreak. Total RNA was extracted in accordance with He’s protocols and sequencing was performed by using a random approach on the short-read Illumina MiSeq platform (Illumina, San Diego, CA 92122 USA). Eight nearly complete genomes and two partial genomes were obtained from 11 samples. Complete coding sequences from the genomes were used to build phylogenetic trees. The completed coding sequences produced a more accurate phylogenetic resolution than that otherwise obtained using partial sequences. The technology allowed the identification of two distinct lineages of sub-genotype VIIi NDV circulating in Pakistani poultry farms.

De Oliveira (2021) analyzed the association of runting and stunting syndrome-associated histopathology with the presence of a viral agent in paraffine-embedded chicken samples of different ages [30]. Tissue sections were collected, and total RNA was extracted after deparaffinization and used for quantification and RNA quality checks. DNA libraries used for random NGS sequencing were prepared using the KAPA single-stranded RNA-Seq Library Preparation Kit for Illumina platforms. Sequencing was conducted on an Illumina MiSeq instrument using a 300 cycle and the raw sequencing data were analyzed using the Galaxy platform interface [31,32]. Analysis of sequencing data detected the presence of avian nephritis virus, avian rotavirus, and picornavirus in jejunal segments from 7-day-old chicks. Detection of picornaviral reads was significantly associated (*p* < 0.05) with histologic lesions of runting and stunting syndrome.

Short-read platforms were also used for simultaneous non-targeted discovery of unsuspected agents and characterization of novel genetic diversity of Newcastle disease virus in fresh or frozen samples. Significant advances were made in 2017 by Dimitrov and collaborators by optimizing a method for the random sequencing of RNAs from chicken eggs allantoid fluids [12]. Dimitrov used NGS-based random sequencing of total RNA combined with barcoding for the simultaneous sequencing of multiple libraries. This approach allowed quick and simultaneous characterization of the genomic sequences of different avian viruses and co-infecting agents. Thirty libraries were prepared from diagnostic samples amplified in allantoid fluids and their total RNAs were simultaneously sequenced in a single flow cell on an Illumina MiSeq instrument. A customized workflow was used to analyze the data on the freely available Galaxy platform, and as a result a total of twenty-eight avian paramyxovirus 1 (APMV-1), one APMV-13, four avian influenza and two infectious bronchitis virus complete or nearly complete genome sequences were obtained [31,32]. Additionally, the simultaneous sequencing of ribosomal RNA present in those samples allowed the detection of novel bacterial species [12,33]. The process proved to be time-efficient and cost-effective as sample processing and library preparation were achieved in approximately 25–30 h, the sequencing run took 39 h, and processing via the Galaxy workflow took approximately 2–3 h. The cost of all steps, excluding labor, was estimated to be approximately USD 106 per sample. The multiplexing of samples for NGS with the Galaxy workflow platform resulted in an effective protocol for the simultaneous characterization of multiple full-length viral genomes. Similar work was conducted by the Van Borm team in 2021 [34]. The Borm team tested the short-read random primed approach for sequencing in two IBV-positive clinical samples and one in an ovo-passaged virus and obtained complete genome assemblies with 99.95% identity to reference strains. In addition, the team describes the assembly of a near-complete chicken astrovirus genome and the presence of chicken calicivirus and avian leukosis virus from the same samples.

Novel genetic diversity was discovered when deep sequencing revealed the existence of non-expected genomic information which was not available via RT-PCR. Kariithi and his team, conducting surveillance in Kenya, identified avian influenza and new strains of Newcastle disease virus sub-genotype V.3 in indigenous chickens from backyard poultry farms and from live bird markets in Kenya [35,36]. Youk and collaborators discovered isolates and characterized the evolution of highly pathogenic H7N3 and H5N2 avian influenza viruses in poultry using NGS [37,38]. Sabra and collaborators utilized NGS to obtain a more precise phylogenetic classification of the circulation of pigeon-derived virulent avian avulaviruses 1 in Eastern Europe, Asia, and Africa [39]. Diversity was obtained in circulating infectious bronchitis viruses of the strain DMV/1639 of the GI-17 lineage [40] and circulating Newcastle disease viruses from Indonesia also by utilizing random sequencing approaches on the Illumina Miseq platform [41]. Kariithi, utilizing clinical swabs of individual chickens from backyards and markets in Kenya [35], unexpectedly discovered and obtained genetic characterization of the first H9N2 low pathogenicity avian influenza viruses isolated from chickens in live bird markets. The team extracted cloacal and respiratory swabs and used the frozen samples to conduct random NGS to amplify nucleic acids with a sequence-independent, single-primer amplification (SISPA) protocol, followed by short-read sequencing on an Illumina MiSeq platform using the 500-cycle MiSeq Reagent Kit v 2. Goraichuk. Collaborators discovered five novel siciniviruses from poultry samples from North America [42]. In a different case, a 15-year-old sample confirmed the circulation of the GA08-like strain and complete genomes of avian coronavirus 4 years before its first reported outbreak [43]. Other agents identified and completely characterized at the genome level by Goraichuk include avian paramyxoviruses of serotype 10 [44], NDV of subgenotype VII.2 from Indonesia [41], fowl aviadenovirus D [45], and three chicken parvoviruses [46].

Mixed bacterial and viral infections were repeatedly found in samples from mucosal tissues. Mixed viral infections, together with multiple bacterial, fungal, and eukaryotic agent combinations, are known to be commonly present in clinical samples [47,48,49]; however, a better understanding of infection patterns in clinical cases was missing. Using a similar approach, Sharma and others discovered and further characterized the complete genome of an unexpected bacterial agent present in multiple NDV-positive samples from chicken samples from Nigeria and Pakistan. The new bacteria carrying multiple antibiotic resistance genes was a new variant of *Ochrobactrum* spp., isolated from different avian hosts [50,51]. The bacteria were discovered using random libraries prepared using the Nextera XT DNA library preparation kit and Nextera XT index primers (Illumina, San Diego, CA, USA). The complete genomes of these bacteria, including the antibiotic resistance genes, were later sequenced using random short-read sequencing by the same authors.

The direct use of genomics to support field surveillance was previously proposed by others [7,34]. The active monitoring of chicken farms for diseases presents unique challenges compared to laboratory research or mammalian work with single animals. These challenges include technical complexities and cost issues during sampling and transportation. It is not currently economically practical to sample individual birds, except in cases where they represent the health status of the entire farm, due to the low cost of birds. Since most commercial poultry systems today consist of farms with millions of birds, it is however theoretically possible to develop affordable sampling methods that are representative of farm health conditions while achieving a good representation of the agents present. The development of acceptable and reproducible sampling protocols made the implementation of NGS-based monitoring at the farm-wide scale reliable and economically feasible. Recently, short-read sequencing has also been used for the discovery of disease-causing agents during active NGS surveillance of Latin American farms [15,25].

This program, which emerged because of a research collaboration to identify circulating disease agents in Latin America, has been able to overcome some of those difficulties. The program was a collaboration between the USDA Southeast Poultry Research Laboratory (SEPRL) in Athens, Georgia, the company Boehringer Ingelheim Animal Health Inc., Duluth, GA 30096 USA, and Base2Bio LLC, Oshkosh, WI 54904, USA. The technology involved three steps or phases. Phase one was the collection of samples from across Latin American countries and the shipment of nucleic acids to the USDA SEPRL laboratory. For sample collection, the team developed standard operating procedures. Swabs from 25 to 100 individual animals per farm were placed in PBS solution and 100 microliters were spotted on FTA cards (Whatman cards). Cards were shipped in envelopes at room temperature to the USDA sequencing lab for sequencing on an Illumina Miseq sequencer. The second phase involved sequencing at the USDA lab. A total of 48 samples (farms) were multiplexed per run. These were sequenced in an Illumina Miseq and the raw data were transferred to Base2Bio for data analysis and reporting. The third phase involved data processing, analysis, and reporting. Reports were presented in an interactive format in individual files and sent via a link to the originator. Each of the 48 libraries produced approximately 350,000 to 500,000 read pairs, each 150–300 nt in length. Reports were produced with graphical interfaces, tables of identified taxa, extractable sequences, and partial analysis of the taxa of interest, including multiple alignments and phylogenetic trees. Data analysis was also performed within Galaxy and Geneious Prime [52,53] at SEPRL to confirm the results.

As of April 2022, 785 clinical samples from commercial poultry flocks in Latin America were collected and analyzed using non-targeted short-read NGS and with a cost of reagents (including sequencing and analysis) ranging from USD 200–350/sample. Samples from 8 countries were analyzed as follows: Mexico (637 samples), Colombia (27 samples), Brazil (*n* = 24), Peru (*n* = 23), Argentina (*n* = 19), Chile (*n* = 19), Ecuador (*n* = 18) and Guatemala (*n* = 18). The type of samples were respiratory (*n* = 405), immunological (*n* = 278), cloacal (*n* = 64) and other tissues (*n* = 38). The most significant viral taxa identified were, in order of frequency of occurrence, sicinivirus, infectious bronchitis virus, infectious bursal disease virus, avian orthoavulavirus, avastrovirus, rotavirus A, influenza A virus, megrivirus, avian metapneumovirus, rotavirus D, rotavirus F, avian orthoreovirus and gallivirus, plus gyrovirus, phacovirus, fowlpox virus, gallid alphaherpesvirus 2, chicken picornavirus 1, chicken picornavirus 5, fowl aviadenovirus D, rotavirus G, and tremovirus A in 3 or fewer samples. Similarly, identification of key disease-causing bacteria was performed. Bacteria included *Enterococcus cecorum*, *Gallibacterium*, *Streptococcus pluranimalium*, *Enterococcus faecalis*, *Bordetella avium*, *Enterococcus faecium*, *Ornithobacterium rhinotracheale*, *Campylobacter*, *Salmonella enterica*, *Avibacterium*, *Enterococcus hirae*, *Enterococcus durans*, *Mycoplasma synoviae*, *Mycoplasma gallisepticum* and *Streptococcus gallolyticus* among others.

Peer-reviewed manuscripts were published describing specific discoveries resulting from this project [15,25]. In [15], the authors used random ntNGS surveillance on clinical samples to report the existence of new variants of infectious bronchitis viruses from commercial flocks in Mexico during 2019–2021. The viruses belonged to the different lineages GI-1 (Mass-type; *n* = 8), GI-3 (Holte/Iowa-97; *n* = 2), GI-9 (Arkansas-like; *n* = 8), GI-13 (793B; *n* = 14), and GI-17 (California variant; CAV; *n* = 1). Point mutations, substitutions, insertions, and deletions were found in the S1 hypervariable regions (HVRs I-III) across all viruses. Also, intra/inter-lineage recombination events were detected in the S proteins. Interestingly, the study demonstrated that FTA cards could be used on field-collected clinical samples to obtain high-quality genetic information from RNAs for the untargeted discovery of avian viral agents. In [25], the Karithii team reported the detection and genome sequence analysis of avian metapneumoviruses circulating in commercial chicken flocks in Mexico by utilizing the approach of direct random sequencing of clinical samples [25,54]. The identification of 11 complete and 2 nearly complete genome sequences of aMPV-A from Mexico was reported. The Mexican aMPVs were closest to the UK strain turkey/UK/8544/2006 for both the genome (96.76–97.48% nucleotide identity) and attachment (G) gene sequences (95.1 and 95.8%), respectively. Using sequence data from this study, the authors revised a previously published RT-PCR test, resulting in a test that was more compatible with other commonly used RT-PCR diagnostic cycling conditions. This was the first comprehensive genome sequence analysis of aMPVs in Mexico and demonstrated the value of using nontargeted NGS to identify pathogens in farms where targeted surveillance for aMPVs is not performed.

## 4. Long-Read Sequencing on Poultry and Avian Species

Long-read sequencers (e.g., Oxford nanopore (ON), https://nanoporetech.com/, accessed on 24 November 2023 and PacBio, https://www.pacb.com/, accessed on 24 November 2023) have been preferentially used for assemblies of large genomes because the longer reads produced are more effective at assembling repetitive regions present in large genomes, despite the higher error rate in comparison to short-read sequencers. Other advantages of some of the long-read ON family of sequencers over other short-read sequencers such as the Illumina platforms are speed from sample to results, low cost, and portability. Of those advantages, perhaps the most important from the diagnostic point of view is the capacity to run fewer samples at a lower per-run cost in some of the smaller sequencers (e.g., the MinION sequencer) [55,56,57,58]. Another advantage over short-read sequencers is derived from rapid output (allowing immediate analysis after run initiation, also called real-time sequencing), which makes them more adaptable to rapid diagnostics. Early access to data from sequencers with rapid outputs could also enable more timely responses, such as the imposition of movement restrictions and quarantine zones by management agencies.

Long-read sequencing is a rapidly evolving technology with numerous applications. The long-read approach appears to be suitable for targeted diagnostics because it typically provides shorter sample preparation protocols, shorter run times, real-time sequencing analysis (during the run), and lower cost per run; however, sequencing technologies continue to evolve and it is hard to predict which technology will dominate the market in the future. The most widely used long-read tNGS test in the world and the proof of the principle of the wide-scale applicability of the technology is the ARTIC protocol, used for the characterization of circulating variants of SARS-CoV-2 [59,60,61]. tNGS methods using long-read sequencers have also been used for the rapid genetic characterization of other known disease agents present in clinical samples [58,62,63,64]. As usually happens, the technology was developed first for those agents that are more likely to have a larger customer base; thus, the development of experimental protocols for long-read tNGS sequencing of the most significant avian diseases, such as Newcastle disease viruses, avian influenza, infection bronchitis, and infectious laryngotracheitis, preceded that for other agents.

Like RT-PCR or Sanger sequencing, a hypothesis is needed in tNGS for the design of primers that amplify nucleic acids from a suspected target agent. This approach is well suited for avian influenza, a segmented virus in which all fragments have similar terminal ends amenable to universal priming. Here, NGS may replace RT-PCR as this last approach does not provide the specificity or the richness of information provided by sequencing. The nucleotide sequences produced via NGS provide rapid specific genotypic and virulence information. This contrasts with RT-PCR, in which only positive–negative results are obtained when additional controls are incorporated into the assay. The older Sanger sequencing approach provides some of the sequence specificity that is missing in RT-PCR, but the low throughput of this technology makes this approach a costly and outdated solution. In addition, Sanger-obtained sequences do not offer the option of separating reads and resulting sequences are a consensus of multiple reads. Thus, there is limited information on the presence of genetic variants, mutants, or mixed infections.

A diverse group of authors has described the use of long-read sequencing to obtain complete genomes of avian influenza [62,63,65]. This virus, which has zoonotic potential and the capacity to reassort and rapidly mutate, is a typical example of why it is important to achieve full genome characterization to predict possible phenotypes. Most of the developed methods had in common rapid and high-throughput workflows. These were based on a universal set of primers that target a region conserved at the end of all 8 AIV genes. However, in the method used by Butt [65], ntNGS (random sequencing) was used for avian influenza. Results were obtained using either one- or two-step reverse transcription PCR amplification reactions, followed by the sequencing of representative samples of different serotypes. In most cases, nucleic acids were directly sequenced simultaneously for all the viruses, using barcoding for multiplexing. The approach produced up to 100%, or in some cases partial, coverage with high confidence in the sequences of all segments, depending on the quality of the RNA samples. The procedure from extraction to results usually takes only hours.

Butt’s team also utilized long-read tNGS to detect virulence and classify clinical samples containing Newcastle disease virus [66]. The author utilized sequential 10-fold dilutions to compare tNGS to RT-PCR and obtained high-quality sequences for all dilutions that were determined positive via RT-PCR, with NDV reads obtained as soon as 5 min after the sequencing run started. At viral concentrations of 10^6^ to 10^3^ EID50/mL, the output produced sequences that had 99.18–100% sequence identity to the reference LaSota strain used to develop the approach. Using clinical swabs, the detection and genetic characterization of virulent and vaccine NDV isolates with long-read nanopore sequencing was performed. The results were comparable to those obtained using short-read Illumina MiSeq sequencing, with no differences observed at the fusion gene cleavage site or in genotype classification when conducting phylogenetic analysis. Most importantly, mixed infections of virulent viruses with live vaccines were detectable. Table 1 (adapted from Table 4 in the original publication) in this experiment shows that both long and short methods can detect mixed infections, but there are still some differences in sensitivity. The same NDV-positive clinical samples that contained one NDV genotype were detected using Miseq and MinION. However, the MiSeq method detected two genotypes in samples #45, #46, #47, and #49; the MinION protocol only detected dual genotypes in samples #45 and #46; while in sample #48, only one NDV genotype was detected using MiSeq and two were uncovered via the use of MinION. The average time (including sequencing time) needed to analyze the 33 samples was approximately 26 person-hours, with USD 31 spent in reagents per sample. This long-read sequencing protocol allowed rapid and accurate detection, virulence determination, and genotyping for clinical swab samples with preliminary analytical sensitivity comparable to that of the matrix RT-qPCR test.

Real-time MinION-based amplicon sequencing for lineage typing of infectious bronchitis virus (IBV) from upper respiratory samples was also reported by Butt in [56]. Here, the author aimed to replace Sanger sequencing of the S1 subunit of the spike gene to characterize and genotype IBV isolates. By developing an amplicon-based sequencing method, IBVs from clinical samples were genetically characterized, including those from samples containing multiple mixed IBV isolates. An additional improvement was that the amplicons were barcoded by design to allow for the pooling of samples. This method detected IBV in 13 of 14 RT-PCR samples and was able to differentiate the lineages, demonstrating the feasibility of using MinION-based sequencing for rapid and accurate genotyping using oral swabs from chickens.

Similarly, for a large DNA virus, a procedure that simultaneously allowed the characterization of the genotypes of US strains of infectious laryngotracheitis virus was developed by Spatz and others for use in the MinION nanopore device [57]. These authors developed a single-allele genotyping system based on examining single-nucleotide polymorphisms (SNPs) within a genomic region that was amplified and sequenced. The ON technology was used for the rapid and simultaneous sequencing and genotyping of multiple ILTV strains. The products of multiple ILTV strains were barcoded individually and sequenced together in a single MinION run.

**Figure 1:**
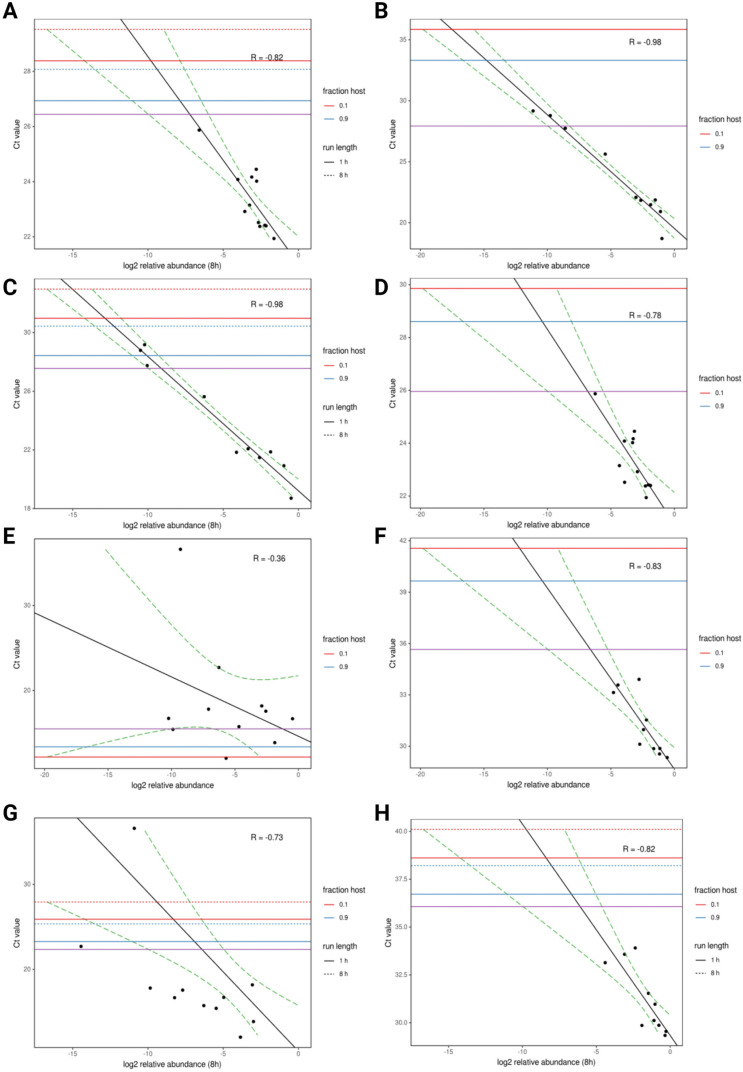
(excerpted from Figure 1 in [65]). MinION and MiSeq read abundance vs. RT-PCR. MinION and MiSeq read abundance vs. RT-PCR Ct; for avian influenza virus (**A**,**B**); for infectious bronchitis virus (**C**,**D**); for *Mycoplasma synoviae* (**E**,**F**) in experimental samples; and for Newcastle disease virus (**G**,**H**) in clinical samples. Black line indicates best-fit linear regression model. Red/blue horizontal lines mark Ct thresholds at which a single read would be estimated to be observed with 95% confidence at different run times and levels of host contamination, given current experimental conditions and assuming 12 multiplexed samples. Purple horizontal line marks Ct threshold, corresponding to one agent on average.

Long-read platforms have also been used for the rapid non-targeted and simultaneous discovery of unsuspected agents and novel genetic diversity in fresh or frozen samples. To investigate the applicability of long-read sequencers for random sequencing, the ability of long- and short-read untargeted sequencing approaches to rapidly detect the viral and bacterial pathogens co-infecting clinical samples was compared [65]. A random sequencing approach based on the MinION platform was used on oropharyngeal swab samples from chickens, experimentally infected with infectious bronchitis virus (IBV), avian influenza virus (AIV), and *Mycoplasma synoviae* (MS), and from field-collected chicken clinical oral swab samples (*n* = 11) taken from live bird markets. Total RNA was randomly reverse-transcribed, amplified, and barcoded, and double-stranded cDNA libraries were pooled for sequencing. For comparison, RNAs from the same samples were also randomly sequenced on an Illumina MiSeq sequencer. Microbial reads were detected within 30 min of MinION sequencing and accurately classified on the basis of taxon and, in many cases, genotype. Working from the experimental swabs containing viral and bacterial agents, MinION relative read counts for each agent (AIV, IBV, and MS) correlated with the RT-qPCR Ct values from all twelve samples (Figure 1, adapted from Figure 1 from [65]). The authors determined the relative read abundance produced by ntNGS and used it to understand the relationship between NGS and RT-qPCR in terms of the detection of viral and bacterial samples (Figure 1A–H). In this figure, a model was fit to the data, and the lowest Ct value at which an agent read would be detected was estimated. The estimated Ct thresholds at which a single read (MinION/MiSeq sequencing) per thousand microbial reads is to be observed with 95% confidence (assuming 12 multiplexed samples per run) ranged between 27 and 27.5 for AIV, 26.5 and 26 for IBV, and 36 and 36.5 for MS. For the three agents used in the study, there was a strong correlation between Ct and log2 abundance (between −0.82 and −0.98). IAIV correlation was strongest, (Figure 1A,B) followed by IBV (Figure 1C,D) and MS correlation (Figure 1E,F). In the field-collected clinical samples, both the MinION and MiSeq approaches detected Newcastle disease virus (NDV) in all RT-PCR-positive samples. In some clinical samples, coinfection with additional respiratory bacteria was detected. Random nanopore sequencing thus provided an alternative rapid cost-effective way of detecting respiratory pathogens. Most significant, the existence of a strong correlation between number of reads and Ct values predict that it should be possible to determine the sensitivity of the approach for each agent accurately if a standard operating procedure is followed. Different technologies have different niches; however, the field is constantly evolving. The applications for short- and long-read random sequencing approaches are numerous and complementary, including the rapid and precise characterization of pathogens; therefore, usage will likely be optimized and adapted in the upcoming years to satisfy the different types of market need.

## 5. Direct Short and Long-Read Sequencing for Applications Not Related to Avian Disease Diagnostics

Short-read sequencing has also been used for the characterization of the effects of nutrition and management on the microbiota [67,68]. Age, nutrition, antibiotics, and bacterial colonization are factors that affect the microbiota of chickens, leading to changes in food efficiency as well as differences in responses to exposure to disease and zoonotic agents. A large body of work describes the multiple applications of this increasingly large field of research and its field applications [67,68]. Most studies on the relationship between diet and microbiota have been based on either the utilization of targeted sequencing on the 16S variable region of bacterial ribosomal RNAs or total DNA metagenomic analysis. While 16S studies are more affordable, these studies in general do not have specificity at the bacterial species levels. Additionally, they are still capable of detecting changes in the abundance and diversity of bacterial genera [21,22,68,69,70]. Total DNA-based metagenomics provides higher resolution than 16S ribosomal amplicon sequencing for bacteria identification but involves larger, more complex, and expensive protocols. Currently, complex metagenomics projects in general are not pursued at the local poultry farm level but are being developed by large multinational companies as services utilizing machine learning algorithms (e.g., using Cargill Galleon platform for nutrition and DSM Verax technology to study blood biomarkers during nutrition), among others. DNA metagenomics has also been used to track antimicrobial resistance [71,72,73,74,75,76] and new machine-learning algorithms are being developed for chickens and other species [77,78,79,80,81,82,83]. In chickens, multiple studies have used metagenomics for the detection of campylobacter [84,85], the tracheal virome [19], and the effect of diet [86] among others.

Both short- and long-read ntNGS have been used for quality control in bird products and detection of contaminants in biological products and vaccine manufacturing [87]. Similarly, as in farm management, quality control of final products, biologicals, and vaccines normally requires multiple tests for endogenous agents that may be present in cells and media [88,89]. NGS is already being used informally by vaccine companies to check the quality of vaccines and it may not be long before regulatory agencies require comprehensive NGS-based tests for adventitious agents. MacDonald and collaborators describe the use of this approach to detect adventitious agents in vaccines and biotechnology-based medicines [87]. The approach was also previously used for the detection of minority variants and adventitious viruses in attenuated vaccines [88,89]. The NGS-based metagenomics approach in general had the advantage of increasing the speed and accuracy of viral detection in comparison to classical targeted methods.

The presence of zoonotic disease agents in poultry products is of great concern as the late recognition of a problem leads to recalls and lawsuits, with millions of dollars in economic losses. Recently, multiple efforts have been targeted toward the characterization of microbiota in finalized chicken products. Some NGS-based approaches for rapid sample characterization are increasingly being used in food quality laboratories and bioinformatics and machine learning approaches are being developed for automated pathogen and gene detection. The use of NGS to analyze the microbiota present in stored chicken breast [90,91] and in other food products has been reported [92,93,94,95]. Today, food quality laboratories are often equipped with sequencing machines from Illumina or Ion Torrent and portable devices from Oxford Nanopore (ON) for metagenomic profiling and complete genome sequencing. In addition, there are numerous bioinformatics services for the NGS analysis of food-borne pathogens and websites that provide support such as the GenomeTrakr network (https://www.fda.gov/food/whole-genome-sequencing-wgs-program/genometrakr-network accessed on 24 November 2023) and the Evergreen online platform. Additionally, software is constantly being improved for those applications [96].

The presence of bacteria associated with chicken meat spoilage and storage conditions was analyzed by several authors, and associations between different genera (*Acinetobacter*, *Brochothrix*, *Thermosphacta*, *Flavobacterium*, *Photobacterium*, *Pseudomonas*, *Psychrobacter*) were found [90,91,97]. Some companies are offering services [98,99,100] in this area, such as Clear Labs, a business providing fully automated NGS platforms (https://www.clearlabs.com/ accessed on 24 November 2023). The Clear Safety Salmonella test has been approved by the National Poultry Improvement Plan (NPIP) for interim use in the detection of Salmonella. NPIP is part of the United States Department of Agriculture’s (USDA) Animal and Plant Health Inspection Service and aims to safeguard the health of the nation’s agricultural resources. The Clear Safety platform uses an automated next-generation sequencing platform to replace polymerase chain reaction, culturing, and antigen-based methods in order to validate pathogen presence while also providing simultaneous deeper agent characterization, such as serotyping or strain typing. Several agents such as Salmonella, Vibrio, Shigella, Campylobacter, and Listeria, among others, are specifically targeted. Other companies such as QIAGEN (CLC Genomic Workbench) are also starting to develop new NGS products for data analysis-specific applications. The Qiagen QIAseq xHYB NGS-based detection of adventitious agents was designed for the detection of unknown human microbes in biopharmaceutical manufacturing. The system is based on enrichment and it detects 132 separate viral targets of human concern, setting a precedent for the development of similar systems in other species. Hybrid capture enrichment of target sequences from viral genomes and bacterial genome is combined, and an analysis portal is provided by the company to quickly analyze sequence data.

Previously, we described the use of the ON MinION for *Salmonella* and *E. coli* rapid complete genome sequencing to perform the genetic tracking of outbreaks and determination of antibiotic resistance and zoonotic potential [101]. Deep short-read sequencing was used to assemble accurate complete genome and plasmid reference sequences of these bacteria that infect chickens and humans. Taylor and collaborators then simultaneously sequenced the entire chromosome and plasmid of Salmonella enterica subsp. enterica serovar Bareilly and Escherichia coli O157:H7 using a rapid and random sequencing approach coupled with long-read de novo genome assembly. The sequencing run took just four hours and produced full-length genomes with an average identity of 99.87% for *Salmonella* Bareilly and 99.89% for *E. coli* to the respective MiSeq references. This long-read sequencing protocol produced readily available information on serotypes, virulence factors, and antimicrobial resistance genes. Although the procedure described above involved the prior culturing of the bacteria, efforts are being made to avoid culturing by using either DNA shotgun metagenomics or tNGS with amplicons [102,103].

## 6. Challenges to the Adoption of NGS Diagnostics

One of the most significant challenges of NGS sequencing for diagnostics is the high cost. Regardless of high cost, long- and short-read sequencing technologies are making significant advances in clinical applications in avian and non-avian fields [20,102,103,104,105,106,107,108,109,110,111,112,113,114]. Added to the benefits of abundant throughput, long (ON and PacBio)- and short-read (primarily Illumina) sequencing technologies offer many advantages over other classical diagnostics methods that are driving usage. NGS offers the possibility of a richer, more precise, and more informative option than RT-PCR. With new commercial kits adapted to extract different types of nucleic acids, robotic automation during sample preparation, and kits for a low input of nucleic acids constantly being developed by leading companies, cost issues will likely diminish. With improvements in throughput, with increased quality of the output, and with open source and commercial bioinformatics software (Galaxy platforms, Geneious, Qiagen, Base2bio, etc.), significant progress is expected to be made in terms of further reducing the per-sample cost of sequencing. It is possible to envision that targeted next-generation sequencing may be initially able to replace the RT-PCR and Sanger-targeted approaches for rapid diagnostics and later it may expand into ntNGS approaches. Later, once NGS-based diagnostics becomes mainstream and the volume of samples received by commercial sequencing labs increases, the daily use of large-throughput sequencers may allow for the multiplexing of hundreds of samples, leading to the development of new economically competitive applications.

An important challenge is derived from the overall complexity of the entire process, from sample collection to data analysis. Sampling is more complex as sequencing normally requires a larger quantity of nucleic acids and less environmental contamination than PCR or RT-PCR. The transport of specimens from the field to the laboratory is another important limitation that more severely affects those farms located at isolated sites or in countries that do not have laboratories placed locally. Degradation has a more negative effect on sequencing than on RT-PCR because the information is often obtained from longer fragments of nucleic acids. International shipment of samples to specialized laboratories can be a lengthy and complex process that causes delays in obtaining critical diagnostic information. The detection of total viral and bacterial agents present in clinical samples at the local farm level is a method that is likely to be attractive for disease control and production effectiveness; however, the lack of availability of complex laboratories required to process samples and the cost of computer hardware and bioinformatics support currently limits its use to a few leading vaccine companies or large poultry production conglomerates. The ON line of long-read portable sequencers, together with the increasing number of kits designed to sequence clinical samples and offer online bioinformatics support, is likely to enable the ability in a not-so-distant future to conduct sequencing locally, using rapid internet connections to process data and obtain actionable reports within hours of a suspected case. As with the issue of cost, an increase in demand will likely be accompanied by the development of other solutions to the complexity issue. The issue was first addressed by Howson in 2017 [4]. Howson suggested that the potential to conduct rapid and decentralized sequencing tests to detect pathogens will become more and more feasible as new technologies emerge.

Another key aspect of the adoption of NGS-based diagnostics is the development of standard operating procedures and the creation of methods for the validation of protocols. The problem has been previously addressed in several reviews as it is as common a problem in poultry as it is for humans and other mammals [2,105,115,116,117,118,119]. The authors recognize the potential diagnostic value of NGS for the control and management of infectious diseases and the need to include and address existing validation and quality control principles and methods accepted by the World Organization for Animal Health and other organizations. Borm discusses the need to normalize the steps involved in the generation of sequencing workflows for veterinary infection studies and suggests that NGS initially may be used as an adjunct diagnostic method, before becoming a primary diagnostic method. In addition, Borm and his team also evaluate the issues that arise with the management and use of massive amounts of information obtained from high-throughput technologies. Besides the immense value of the information that is used for the molecular identification and characterization of agents, NGS also provides numerical information on the microbial community’s composition, viral evolution, and host transcriptome (not developed yet into a diagnostic discipline). The complexity of the development of standard operating procedures and validation at the laboratory level has the potential to further increase with the added difficulty of analyzing and interpreting bioinformatics data for multiple agents simultaneously. Complexity is expected to increase in the future as biological networks are detected and agent-to-agent interactions are detected in some of the complex diagnostic pipelines produced by random sequencing.

One key aspect in the development of standard operating procedures is sampling.

As the first step, sampling is key because it cannot be repeated exactly if not performed appropriately. Furthermore, the well-known principle (garbage in = garbage out principle) is more applicable to ntNGS than to other classical diagnostics methods. Due to its complexity and high cost, sampling for NGS implies that development of good sampling SOPs cannot be ignored or corrected with excellent post-sampling processing or bioinformatics. Thus, multiple additional sampling aspects need to be respected and further developed to obtain reliable results with NGS (Table 2). Table 2 describes the key aspects to consider during sampling.

The issue of the validation of each step for NGS application has been reviewed by Halpin recently [120]. Validation includes addressing the issues of complexity of data extraction, complexity of analysis, and interpretation of data. The author describes that the validation challenges involve a multiplicity of aspects, such as sequencing platform validation, wet lab process validation, and bioinformatic pipeline validation. In addition to the normal quality controls, proficiency testing and the use of reference materials to obtain the desired performance metric of diagnostics will be needed. Performance metrics such as the frequency of true positive and false positives, sensitivity and specificity, repeatability, and limits of detection, are among the challenges ahead. However, the authors conclude, that “while the employment and subsequent validation of a new technology is always going to be challenging, there are existing guidelines and frameworks, checklists and international standards that can be used”. Of course, since NGS is a relatively new technology in this field, there are no validated protocols or standard operating procedures that can be universally adopted in avian species or any other species. The development of nucleic acid extraction, sequencing kits, and data analysis software is continuously evolving to better address metagenomic data generation. The adoption of kits and software from leading manufacturers will likely contribute to the process of validation. There are no immediate solutions in place to address this difficult issue; however, it is expected that the rigorous scrutiny of human medicine diagnostics is likely to lead to solutions that may be transferred in the future into avian medicine.

The development of new approaches to increase the sensitivity of ntNGS is another challenge in terms of extending the applicability of this technology. This issue was first addressed by Rosseel and collaborators in 2015 when they evaluated pretreatment protocols for RNA virus metagenomics in serum and tissue samples [121,122]. The group evaluated the sensitivity and the introduction of bias that may happen upon enrichment or amplification steps, which are widely used for RNA virus discovery. The authors found that enrichment procedures had a positive effect on the discovery of viral agents; however, sample-dependent guidelines may be needed. Newcastle disease virus, rotavirus G, duck hepatitis B virus, and avian leukemia virus were detected in several farms, with results in agreement with RT-PCR assays. Recently, Parris and Suarez described a poultry-specific approach that was developed at the SEPRL labs of the USDA to increase levels of detection of viruses and bacterial detections during non-targeted, high-throughput sequencing. The approach was based on reducing the levels of contaminant ribosomal RNAs from the host and other non-target bacterial sequences during library preparation [123]. An improvement in sensitivity was achieved with reductions in host RNAs, ranging from up to 40% of total reads to as low as 3% in respiratory samples, with, in some cases, up to 700-fold increases in the number of viral reads. The authors report that this custom depletion approach had an added cost of only USD 7–12 per sample. Several research labs continue to work on the issue of depletion of ribosomal depletion to increase sensitivity [123,124,125].

The development of a new generation of biologists, medical doctors, and veterinarians with the capacity to take advantage of these new approaches is another challenge at the policy or educational level. There is no doubt that more qualified professionals are needed to use and develop the full potential of this technology. Currently, the complex results obtained by ntNGS, by which dozens of pathogens are identified from a single sample, have the potential to confuse the veterinarian who is inexperienced in interpreting this type of data. In this context, the presentation of the data can be just as important as the analysis. It is important to develop tools that facilitate the interpretation of the data, such as “per-agent” probabilistic scores for the presence of one or another agent under healthy-sick conditions. The natural conclusions derived from Koch’s postulates, which have been the standard for establishing the microbiological etiology of infection and disease, may also need to be re-interpreted. For example, the well-known fact that one disease agent is usually responsible for an outcome may require the development of new paradigms for the understanding of the pathology of diseases as agent-to-agent interactions, or opportunistic infections, may emerge as causatives of clinical outcomes. This may be more noticeable in mucosal respiratory infections, with multiple agents are more frequently found there than in internal tissues.

The complexity of the interpretation of NGS data may extend beyond bioinformatics and into epidemiology. In infectious diseases, there is normally a pattern of events that starts with infection, development of clinical signs, associated complex infections, and transmission. This process, which may occur in a reduced environment, usually precedes an outbreak. The timing of sampling during these processes is likely to affect the NGS results. The earlier the recognition of the associations between NGS results and clinical manifestations associated with pathology or transmission, the higher the chances of stopping a disease or a chain of transmission that may lead to irreparable damage. However, similar issues have been raised in other mammalian, human, and environmental studies [2,105,117,119,126,127,128,129,130]. The combination of multiple reports in large regional or temporal databases for further epidemiological analysis is likely to have predictive power that could be used in poultry disease prevention and in operation management decisions. All the above are understandably complex problems that are likely to be resolved slowly as enough users discover the benefits of sequencing, and dominant research laboratories and companies develop software for data analysis and superior standards for interpretation that are adopted universally.

## 7. Future Developments

Academia and commercial companies are recognizing the potential of artificial intelligence (AI) to automatically process and manage data and are starting to develop software targeted towards specific applications. Bioinformatics and AI are complementary technologies that may combine to reduce complexity in data handling, analysis, and interpretation. NGS sequencing reports, which can be produced in a universal format amenable as an input to AI algorithms, have the potential to advance in the development of a framework for automated diagnosis, the prediction of outcomes, and the analysis of large-scale trends. Currently, some companies (https://www.basebio.com/ accessed on 24 November 2023) have started this process by automatically producing reports that are easy to use and interpret, while providing fast, extractable information for additional use in AI diagnosis or automation. Their goal is to develop rapid, low-cost, high-throughput NGS pipelines that produce “diagnostic reports”. Ideally, those would be interactive and designed to be rich in information, self-contained, and user-friendly, while providing access to raw reads for further analysis by experienced users.

Automated monitoring, as a warning system against the presence of disease agents, is a desirable feature of diagnostics that may be implemented in the not-too-distant future. Healthy vaccinated animals can harbor unnoticed poultry disease-causing and zoonotic agents, and early detection using active surveillance may allow for preventive or early mitigation actions to avoid significant economic damage. The atomization of viruses, creating virus-containing droplets and aerosols, is one aspect amenable to automated disease detection as the presence of airborne viruses is normally followed by infection. The use of farm or chicken dust automated collecting devices has the potential to eliminate the tedious work of swabbing multiple animals and may be used to verify or validate safety and biosecurity procedures. Preliminary work suggests that it is possible to capture viruses from dust and detect them via sequencing [131]. Farm dust, used for viral metagenomic surveillance, has allowed the identification of multiple viral agents such as parvoviruses, picornaviruses, caliciviruses, and astroviruses, among others [23,132]. More recently, commercial devices with automated sampling are starting to be marketed to eliminate the manual handling of animals. One of those devices has already proven to be useful for the collection and transport of nucleic acids and has been demonstrated to be capable of detecting infectious bronchitis viruses (https://aerocollect.dk/wp-content/uploads/2022/06/AeroCollect_Application-note-IBV_Indical_EN.pdf, 24 accessed on November 2023).

Multiple futuristic ideas suggest that AI and disease tracking may be integrated, as described by Broza [133]. The author explores various solutions for rapid detection that he proposed during pandemic crises. Bio-diagnostics, based on animal temperatures or chemical and electrochemical sensing devices triggering a request for NGS data analysis with AI, machine learning, and clinical support, could improve the accuracy of predictions of patterns indicative of disease states. Many machine learning applications to aid in bioinformatics, analysis of biological network analysis of microbiomes, taxonomic and functional annotations, and clinical diagnosis, are already being explored, including one study that assesses the risk of Salmonella contamination in poultry samples [80,83,134,135,136,137]. Undoubtedly, machine learning applications have the potential to contribute to poultry diagnostics when this technology and NGS data analysis become validated.

Baugher recently studied the urinary microbiome, using AI to enhance infectious diagnosis, and Lin proposed the use of AI to understand the metagenomics of intestinal disease diagnostics [138,139]. Machine learning has been utilized recently to study the gut microbiome for the diagnosis of bowel diseases in humans [140,141,142]. In Manandhar’s work, a gut microbiome-based supervised machine-learning approach was used for clinical diagnosis. The hypothesis was tested by comparing the fecal 16S metagenomic data of 729 subjects with bowel disease to the data of 700 subjects without it. Five different machine learning algorithms were compared to detect differential bacterial taxa using linear discriminant analysis and linear discriminant analysis with training using random forest. The study detected increased levels of Firmicutes and decreased levels of Bacteroidetes in subjects with bowel disease. The study demonstrated the promising potential of AI via supervised ML modeling for the predictive diagnostics of bowel disease using gut microbiome data.

Developing algorithms to further facilitate the interpretation of the results of NGS metagenomic information is one AI application that promises to have utility in poultry medicine [134,138,140,143,144,145,146,147,148]. Although sequencing technology produces rapidly growing amounts of data, and although the data are accompanied by the potential to establish biologically relevant associations with use in diagnostics, such studies are in their infancy and most progress has been made in human clinical diagnostics, especially analysis of images. However, AI is starting to develop algorithms for metagenomic analysis and clinical diagnostics in human medicine that may one day be adopted by veterinary applications, including avian disease diagnosis. Prifti recently introduced Predomics, a machine-learning approach inspired by microbial ecosystem interactions that aims to find predictive signatures based on the cumulative abundance of microbiome measurements [149].

An undeveloped but potentially promising aspect of random sequencing of RNAs is the study of data related to host expression. It is known that random sequencing of tissue samples infected contains up to 99% of host RNAs, which includes ribosomal as well as messenger RNAs [150,151]. Currently, host sequences are often discarded, but integration of agent discovery and host response analysis can potentially lead to better diagnostics. This is another type of complex analysis that will initially require the development of standard operating procedures, the complete development of software for interpretation, and the development of large databases for use as training sets during the creation of tools used by machine learning algorithms. However, as with all promising human endeavors, progress will be made step by step.

## 8. Conclusions

The future of NGS-based diagnostics for the avian industry is bright. The mass-scale capacity of contemporary large commercial poultry farms provides unique cost advantages for the use of targeted and non-targeted applications at the farm level. The development and validation of standard operating procedures for monitoring, sampling, processing, data analysis, and the determination of sensitivity and specificity for specific viral and bacterial agents represent significant challenges. However, the prospects of improving the overall health of farms, utilizing metagenomic or random analysis conducted with minimal human intervention due to automation and AI, are strong incentives to develop the necessary technology. It is also possible to envision how virus–virus, virus–bacterium, virus–fungus, and virus–protozoon relationships may be associated with the prediction of clinical outcomes or with productivity. These associations are currently being observed and recorded by scientists using ntNGS. In all cases, veterinary schools need to consider deepening the training of professionals in bioinformatics. Field veterinarians are generally not well-trained in the production, use, and interpretation of molecular data and are often unaware of the limitations or benefits of a given technology. This training would include understanding the terminology of genomics and bioinformatics, and the limitations of different approaches that may lead to actionable conclusions, especially if the results are complex. However, it is now a good time for academia and private enterprises to invest in developing the NGS-based tools that will be used in the future.

## Figures and Tables

**Table 1:** Excepted from Table 4 in [66]. Identification and virulence prediction of NDV genotypes in clinical samples collected during outbreaks in 2015 (run 5, 6, and 7).

Sample ID	Miseq Genotypes	MinION Genotypes	ID of the MinION Hit	Reads/ Cluster	Consensus Length	Percent Identity	Fusion Protein Cleavage Site ^□^
44	VIIi	VIIi	chicken/Pakistan/Wadana_Kasur/PNI_PF_(14F)/2015	200	734	100	virulent
45	VIIi **II**	VIIi **II**	chicken/Pakistan/Wadana_Kasur/PNI_PF_(14F)/2015 **chicken/USA/LaSota/1946**	28 **5**	734 **733**	99.31 **96.44**	Virulent **Low virulent**
46	VIIi **II**	VIIi **II**	chicken/Pakistan/Wadana_Kasur/PNI_PF_(14F)/2015 **chicken/USA/LaSota/1946**	10 **17**	733 **734**	99.13 **98.51**	Virulent **Low virulent**
47	VIIi **II**	ND ^d^ **II**	NA ^e^ **chicken/USA/LaSota/1946**	NA **139**	NA **732**	NA **99.32**	NA **Low virulent**
48	**ND** VIIi	**II** VIIi	**chicken/USA/LaSota/1946** chicken/Pakistan/Wadana_Kasur/PNI_PF_(14F)v/2015	**200** 21	**732** 733	**99.59** 99.13	**Low virulent** virulent
49	VIIi **II**	ND **II**	NA **chicken/USA/LaSota/1946**	NA **200**	NA **732**	NA **99.32**	NA **Low virulent**
50	VIIi	VIIi	chicken/Pakistan/Wadana_Kasur/PNI_PF_(14F)/2015	113	734	100	virulent
51	VIIi	VIIi	chicken/Pakistan/Mirpur_Khas/3EOS/2015	200	734	100	virulent
52 ^a^	VIIi	ND	NA	NA	NA	NA	NA
53	VIIi	VIIi	exotic Parakeets/Pakistan/Charah/Pk29/29A/2015	5	726	98.5	virulent
54	NO NDV	NO NDV	NA	NA	NA	NA	NA
55	NO NDV	NO NDV	NA	NA	NA	NA	NA
56	NO NDV	NO NDV	NA	NA	NA	NA	NA
57	NO NDV	NO NDV	NA	NA	NA	NA	NA
58	VIIi	VIIi	chicken/Pakistan/Gharoo/Three_star_PF_(7G)/2015	8	729	99.32	virulent
TN ^b^	NA	ND	NA	NA	NA	NA	NA
EN ^c^	NA	ND	NA	NA	NA	NA	NA

^a^ After bead purification, the barcoded amplicon concentration of this sample was lowest in this pool. ^b^ Template control negative. ^c^ Negative extraction control. ^d^ Not detected. ^e^ Not applicable. ^□^ The fusion protein cleavage sites did not vary between AmpSeq and previous MiSeq. Note: Isolates known to have low virulence are highlighted in bold.

**Table 2:** Basic principles of sampling for non-targeted sequencing during active surveillance of poultry farms.

1. Always use “standard operating procedures” for sample-to-sample comparison purposes.
2. Develop “a priori” a sampling strategy focused on the specific problem with the help of a field veterinarian and pathologist.
3. Develop a sampling strategy that covers “completely and evenly” the areas or the host of interest.
4. Minimize contamination from operators, non-target tissues, and from the environment at all stages of collection.
5. Minimize post-sampling contamination; use masks, sterile plasticware, media, and antibiotics if possible for manipulation and storage.
6. Do not mix different types of samples (e.g., cloacal samples will dilute respiratory samples with bacterial nucleic acids).
7. Obtain sufficient starting sample material (RNA/DNA) to minimize the amplification steps (e.g., pool the same type of samples if necessary).
8. Minimize degradation of nucleic acids (RNAs are very sensitive) by using gloves, cold chains, and RNAse-free reagents.
9. Use trained operators at all stages of the process.
10.Use fast and reliable labeling (printed tags, barcoding, spreadsheets, instead of pens at the site).
11. Obtain and link the most complete metadata possible in all samples (e.g., farm clinical and management information).
12. Note “all’ clinical details associated with the host pathology for each individual sample.
13. When spotting on FTA cards, rigorously follow the recommendations on expiration dates, spotting volumes, drying time storage, and shipment conditions.
14. Include information in “the shipping form” that will be used for the interpretation of complex results such as:
Date of collection, the name of the operator, and/or sample contact information.
Flock identification (can be coded for confidentiality)
Type of sample (oropharyngeal, cloacal, tissue).
Species and age of the sampled birds.
Optional information: vaccination; suspected disease; clinical lesions; histology; flock health; production problems; GPS location.

## Data Availability

Data is contained within the article.
